# Reevaluation of surgical strategies for chronic periprosthetic joint infection: a comparison of short-term outcomes between one-stage and two-stage revision

**DOI:** 10.3389/fcimb.2026.1776770

**Published:** 2026-04-13

**Authors:** Qijin Wang, Zhenbao Lu, Jiexin Huang, Jianfu Zhu, Jiliang Chen, Hongkuan Lin, Xiaolu Wang, Jinqing Wu, Qingshan Xu, Wugui Chen, Chengshou Ling, Xu Wang, Xiaodan Lin, Cuihua Yuan

**Affiliations:** 1 Department of Orthopedics, Affiliated Mindong Hospital, Fujian Medical University, Fuan, China; 2 Department of Orthopedic, Affiliated Nanping First Hospital, Fujian Medical University, Nanping, China; 3 Department of Neurology, Affiliated Mindong Hospital, Fujian Medical University, Fuan, China

**Keywords:** bacteria, efficacy comparison, one-stage revision, periprosthetic joint infection, two-stage revision

## Abstract

**Objective:**

This study aims to compare the short-term clinical outcomes of one-stage and two-stage revision surgeries for treating chronic periprosthetic joint infection (PJI), addressing the limitations of the prolonged treatment cycle and heavy medical burden associated with two-stage revision, and evaluating the feasibility and effectiveness of one-stage revision as an alternative therapeutic strategy.

**Methods:**

This retrospective cohort study included PJI patients who underwent surgical treatment at our orthopedic center from July 2018 to November 2023. Patients were divided into one-stage revision group (n=16) and two-stage revision group (n=27) based on the surgical approach. The primary outcome was the infection control rate at 24 months postoperatively, while secondary outcomes included Harris hip score(HHS), Knee Society Score (KSS), and the incidence of postoperative complications within 90 days.

**Results:**

There were no significant differences between the two groups in baseline characteristics. The mean follow-up duration was 29.5 ± 4.5 months. The infection control rate was comparable between one-stage (87.5%, 14/16) and two-stage (88.9%, 24/27) revision (P = 0.89). For hip function, HHS improved from 52.8 ± 6.3 to 85.2 ± 5.1 in the one-stage group and from 55.1 ± 5.9 to 80.4 ± 6.7 in the two-stage group. For knee function, KSS improved from 54.3 ± 6.5 to 84.7 ± 5.8 in the one-stage group and from 56.4 ± 7.0 to 81.2 ± 6.9 in the two-stage group. The one-stage group demonstrated significantly greater functional improvement in both HHS (P = 0.021) and KSS (P = 0.032). Postoperative complication rates were 18.75% (3/16) and 14.81% (4/27), respectively (P>0.05).

**Conclusion:**

In PJI patients with clear preoperative pathogen identification, one-stage revision surgery demonstrates comparable infection control rates and safety to two-stage revision after two years of follow-up, with superior joint function recovery. One-stage revision can effectively avoid a second surgery, shorten the treatment cycle, and reduce patient suffering and economic burden, making it an effective treatment option for eligible PJI patients.

## Introduction

Total joint arthroplasty is a well-established procedure for the treatment of end-stage joint diseases, effectively restoring joint function and significantly improving patients’ quality of life (Neuprez et al., 2020). With the acceleration of the aging population and the widespread adoption of surgical techniques, the number of joint replacement surgeries worldwide continues to rise (Stubnya et al., 2025). However, as one of the major postoperative complications, the incidence of periprosthetic joint infection (PJI) is approximately 1%-2% (Isler et al., 2025). Although this incidence is relatively low, PJI has become a significant challenge in the field of orthopedic surgery due to its complex clinical management, prolonged treatment course, and uncertain prognosis (Nelson et al., 2023).

PJI can lead to catastrophic clinical outcomes, including persistent joint pain, functional impairment, and prosthetic loosening or failure. In severe cases, amputation may be necessary (Nelson et al., 2023). At present, two-stage revision surgery is considered the “gold standard” for the treatment of chronic PJI. This approach involves a staged surgical procedure: first, thorough debridement is performed, followed by the placement of an antibiotic-loaded cement spacer. After confirming infection control, the prosthesis is reimplanted, ensuring a high infection eradication rate (Kini et al., 2016; Hannon et al., 2025). However, this strategy has notable limitations: patients must undergo two surgeries, the treatment course is significantly prolonged, medical costs are substantially increased, and during the interval between the two surgeries, joint function is severely limited, affecting the patient’s quality of life (Kurtz et al., 2022; Zhao et al., 2024).

In recent years, with advancements in precise debridement techniques, one-stage revision surgery has regained attention from orthopedic surgeons. This procedure completes both infection debridement and prosthesis reimplantation in a single surgery. Some studies suggest that in carefully selected cases, the infection control rate of one-stage revision is comparable to that of two-stage revision (Mu et al., 2023; Wignadasan et al., 2023). However, the efficacy of one-stage revision remains uncertain, as its success is highly dependent on clear microbiological evidence, thorough debridement techniques, appropriate prosthesis selection, and standardized perioperative management (Mu et al., 2023; Goh et al., 2025). Therefore, despite the potential advantages of one-stage revision, its clinical application still requires cautious evaluation.

Based on the current research background, this study aims to systematically compare the efficacy and safety of one-stage and two-stage revision surgeries in the treatment of PJI through a retrospective cohort analysis. The study will focus on evaluating the differences between the two groups in infection control rates, joint function recovery, and the incidence of complications, with the goal of providing evidence-based guidance for optimizing clinical treatment strategies.

## Materials and methods

### Study design and participants

This study is a single-center, retrospective cohort study, approved by the institutional ethics committee (K2025121102). The study systematically reviewed the clinical data of patients diagnosed with PJI and undergoing revision surgery at our orthopedic center from July 2018 to November 2023. The diagnosis of PJI was strictly based on the 2018 Musculoskeletal Infection Society (MSIS) criteria (Parvizi et al., 2018). Inclusion criteria were: (1) age ≥ 18 years; (2) diagnosis of chronic PJI (symptom duration >4 weeks) following primary or non-primary hip/knee arthroplasty; (3) preoperative positive culture results**;** (4) undergoing either one-stage or two-stage revision surgery; (5) availability of complete medical records with a follow-up duration of at least 24 months. Exclusion criteria were: (1) uncontrolled systemic sepsis or life-threatening infection; (2) significant bone loss requiring custom or tumor-type prosthetic reconstruction; (3) severe heart, lung, liver, or kidney dysfunction, with a life expectancy of less than one year.

### Surgical methods and perioperative management

Preoperative Preparation: All patients underwent joint aspiration prior to surgery for microbiological culture and drug sensitivity testing. Routine laboratory tests including complete blood count, erythrocyte sedimentation rate (ESR), C-reactive protein (CRP), and joint fluid white blood cell count (WBC) and percentage of polymorphonuclear cells (PMN%) were performed.

### One Stage Revision Patient Selection

The decision to perform a one-stage revision was based on preoperative identification of pathogenic microorganisms and antimicrobial susceptibility testing of the joint aspirate, in combination with an assessment of systemic conditions (normal immune status, absence of poorly controlled diabetes, and no severe soft tissue defects). Patients were informed that two-stage revision remains the international gold standard. The potential advantages of one-stage revision—including avoidance of a second surgery, shorter hospitalization, lower costs, and faster recovery—were also explained. The final decision was made by the patients after thorough discussion, respecting patient autonomy.

### Surgical technique

#### One-stage revision group

Through the original surgical approach, thorough debridement of the sinus tracts and scar tissue was performed, and the original prosthesis and bone cement were completely removed. Extensive debridement included: (1) complete synovectomy; (2) resection of the joint capsule and periarticular scar tissue; (3) removal of all necrotic soft tissue until healthy, bleeding tissue was visualized; (4) placement of iodine-soaked gauze into the wound cavity after debridement, followed by temporary closure of the incision with simple sutures. The surgical site was then re-prepped and draped with sterile towels before proceeding. A pulse lavage system was used for three cycles (3L per cycle), with each cycle consisting of normal saline irrigation, followed by 3% hydrogen peroxide immersion (3 minutes), 0.5% povidone-iodine immersion (3 minutes), and a final saline rinse. A new non-cemented or hybrid fixed prosthesis was then implanted. Antibiotic-loaded bone cement (vancomycin 1g and gentamicin 0.5g per 40g packet) was used for cemented components.

#### Two-stage revision group

First stage: The debridement, prosthesis removal, and pulse lavage steps were identical to the one-stage revision group, including the standardized debridement protocol, iodine-soaked gauze placement with temporary wound closure, re-prepping and draping, and alternating hydrogen peroxide/povidone-iodine immersion cycles. After debridement, an antibiotic-impregnated cement spacer was implanted with vancomycin (2g) and gentamicin (2g) per 40g cement packet. The wound was closed in layers over a closed suction drain. Intravenous antibiotics were administered for 2 weeks, followed by 6 weeks of oral antibiotics, based on culture sensitivities. ESR and CRP were monitored twice weekly. After resolution of systemic/local infection signs and significant decrease in inflammatory markers (ESR <30 mm/h and CRP <10 mg/L), a 4-week antibiotic holiday was initiated.
Two stage: If no infection recurrence was evident during the antibiotic holiday, the spacer was removed. The joint cavity underwent repeat pulse lavage with the same alternating immersion protocol (hydrogen peroxide/povidone-iodine) before implanting a new permanent prosthesis. Intraoperative frozen section analysis (<5 neutrophils per high-power field in 5 separate fields) and multiple tissue cultures were obtained to confirm infection eradication. **And** all surgeries were performed by a single senior arthroplasty surgeon.

### Evaluation indicators

Primary Outcome: The infection control rate after surgery, defined as the absence of PJI recurrence or re-infection during follow-up. Criteria for infection recurrence include deep infection requiring reoperation, sinus tract formation with drainage, or fulfillment of the MSIS diagnostic criteria. Infection status was assessed by a multidisciplinary team consisting of orthopedic surgeons, infectious disease specialists, and clinical microbiologists, who were all blinded to the surgical approach, based on clinical examination, laboratory markers (ESR, CRP), and microbiological cultures.
Secondary Outcomes: **Joint Function**: Hip joint function was assessed using the Harris Hip Score (HHS) (Harris, 1969), which was evaluated by two blinded rehabilitation physicians both preoperatively and at 24 months postoperatively (scale of 0-100, with ≥90 considered excellent, 80–89 good, 70–79 fair, and <70 poor). Knee joint function was evaluated using the Knee Society Score (KSS) (Insall et al., 1989), which includes both function and pain scores, and was similarly assessed by two blinded rehabilitation physicians at both preoperative and 24-month postoperative time points (scale of 0-100, with ≥85 excellent, 70–84 good, 60–69 fair, and <60 poor).

### Complication rate

The incidence of postoperative complications within 90 days, including both surgical and non-surgical complications (e.g., wound issues, hematoma, neurovascular injury, prosthesis instability, etc.), was recorded.

### Statistical analysis

Statistical analysis was performed using SPSS 26.0 software. Normally distributed continuous variables were presented as mean ± standard deviation, and intergroup comparisons were conducted using independent sample t-tests. Non-normally distributed continuous variables were presented as median (interquartile range), and comparisons between groups were performed using the Mann-Whitney U test. Categorical variables were expressed as frequency (percentage), and intergroup comparisons were made using the χ² test or Fisher’s exact test. A p-value of <0.05 was considered statistically significant.

## Results

### Baseline characteristics of the patients

According to the inclusion and exclusion criteria of the study, a total of 47 patients were initially screened. Two patients were excluded due to incomplete clinical data, and one patient was lost to follow-up. A total of 43 patients were included in the final analysis, with 16 patients in the one-stage revision group and 27 patients in the two-stage revision group. As shown in **Table 1**, there were no statistically significant differences between the two groups in terms of age, sex, body mass index (BMI), distribution of affected joints, major comorbidities, and the preoperative positive culture rate and distribution of pathogens (all P > 0.05), indicating that the two groups were well-matched.

**Table 1:** Demographics of patients with chronic PJI undergoing one- or two-stage revision.

Parameters	One-stage revision (n=16)	Two-stage revision (n=27)	P value
Age, yrs (SD)	65.23 ± 7.52	64.85 ± 6.91	0.75^a^
Sex (M)	10	15	0.65^b^
BMI, kg/m² (SD)	25.48 ± 3.29	25.19 ± 2.97	0.48^c^
ASA	2.3 ± 0.5	2.4 ± 0.6	0.67a
Smoking (n)	6	9	0.78^b^
Alcohol use (n)	5	8	0.81^b^
Hypertension (n)	4	8	0.49^b^
Diabetes (n)	5	6	0.77^b^
Sinus tract	2	5	0.35^b^
Joint involved (n)			
Hip	7	10	0.66^b^
Knee	9	17	
Preoperative pathogens			
MRSA	2	3	0.72^b^
Staphylococcus aureus	4	9	0.36^b^
CN Staphylococcus	5	9	0.89^b^
Streptococcus	2	4	0.50^b^
Pseudomonas aeruginosa	1	2	0.45^b^
Other pathogens	2	1	0.63^b^
Median ESR, mm/h (IQR)	50.62 (29.00 to 69.85)	53.58 (26.35 to 75.38)	0.65^c^
Median CRP, mg/L (IQR)	43.28 (15.25 to 72.46)	39.42 (12.37 to 69.57)	0.73^c^
Median SF-WBC, 10^6^/L (IQR)	29385.00 (4827.00 to 51329.80)	33275.00 (3965.00 to 55738.90)	0.82^c^
Median SF-PMN%, (IQR)	81.78 (79.35 to 89.69)	83.45 (78.87 to 91.38)	0.65^c^

^a^ Independent-samples t-test.

^b^ Chi-squared test.

^c^ Mann-Whitney U test.

MRSA, Methicillin-resistant Staphylococcus aureus; CN Staphylococcus, Coagulase-negative Staphylococcus; Other pathogens: Including Corynebacterium striatum, Cutibacterium acnes, Finegoldia magna; ESR, erythrocyte sedimentation rate; CRP, C-reactive protein; SF-WBC, synovial fluid white blood cell; SF-PMN%, synovial fluid polymorphonuclear neutrophil percentage.

### Functional improvement

Both groups showed significant improvements in joint function scores at 24 months postoperatively. In the one-stage revision group, the HHS improved from 52.8 ± 6.3 preoperatively to 85.2 ± 5.1 at 24 months. In the two-stage revision group, HHS improved from 55.1 ± 5.9 to 80.4 ± 6.7. The one-stage group showed significantly greater improvement in HHS compared to the two-stage group (P = 0.021). Regarding knee function, the KSS in the one-stage group improved from 54.3 ± 6.5 preoperatively to 84.7 ± 5.8 at 24 months, while in the two-stage group, KSS improved from 56.4 ± 7.0 to 81.2 ± 6.9. The one-stage group also showed significantly greater improvement in KSS (P = 0.032) (**Table 2**).

**Table 2:** 24-month functional outcomes after one- vs two-stage revision for chronic PJI.

Parameter	One-stage revision (n=16)	Two-stage revision (n=27)	P-value
Preoperative HHS (SD)	52.8 ± 6.3	55.1 ± 5.9	
Postoperative 24-month HHS (SD)	85.2 ± 5.1	80.4 ± 6.7	
HHS improvement (SD)	32.4 ± 6.0	25.3 ± 7.0	0.021^a^
Preoperative KSS (SD)	54.3 ± 6.5	56.4 ± 7.0	
Postoperative 24-month KSS (SD)	84.7 ± 5.8	81.2 ± 6.9	
KSS improvement (SD)	31.4 ± 6.4	23.8 ± 7.5	0.032^a^

^a^ Mann-Whitney U test.

HHS, Harris hip score; KSS, Knee Society Score.

### Complication rate

Within 90 days postoperatively, the one-stage revision group experienced 3 complications, including 2 cases of wound healing issues and 1 case of deep hematoma. The two-stage revision group had 4 complications, consisting of 2 cases of superficial wound infection, 1 case of deep hematoma, and 1 case of spacer fracture. The overall complication rate was not significantly different between the two groups (P > 0.05) (**Table 3**). All complications were appropriately managed and resolved, with no mortality observed during the study period.

**Table 3:** Comparison of complications within 90 days after one- vs two-stage revision for chronic PJI.

Complication type	One-stage revision (n=16)	Two-stage revision (n=27)	P value
Wound Healing Issues	2	0	
Deep Hematoma	1	1	
Superficial Wound Infection	0	2	
Spacer Fracture	0	1	
Total Complications	3	4	0.93^a^

^a^ Chi-squared test.

### Infection control rate

**Figure 1:**
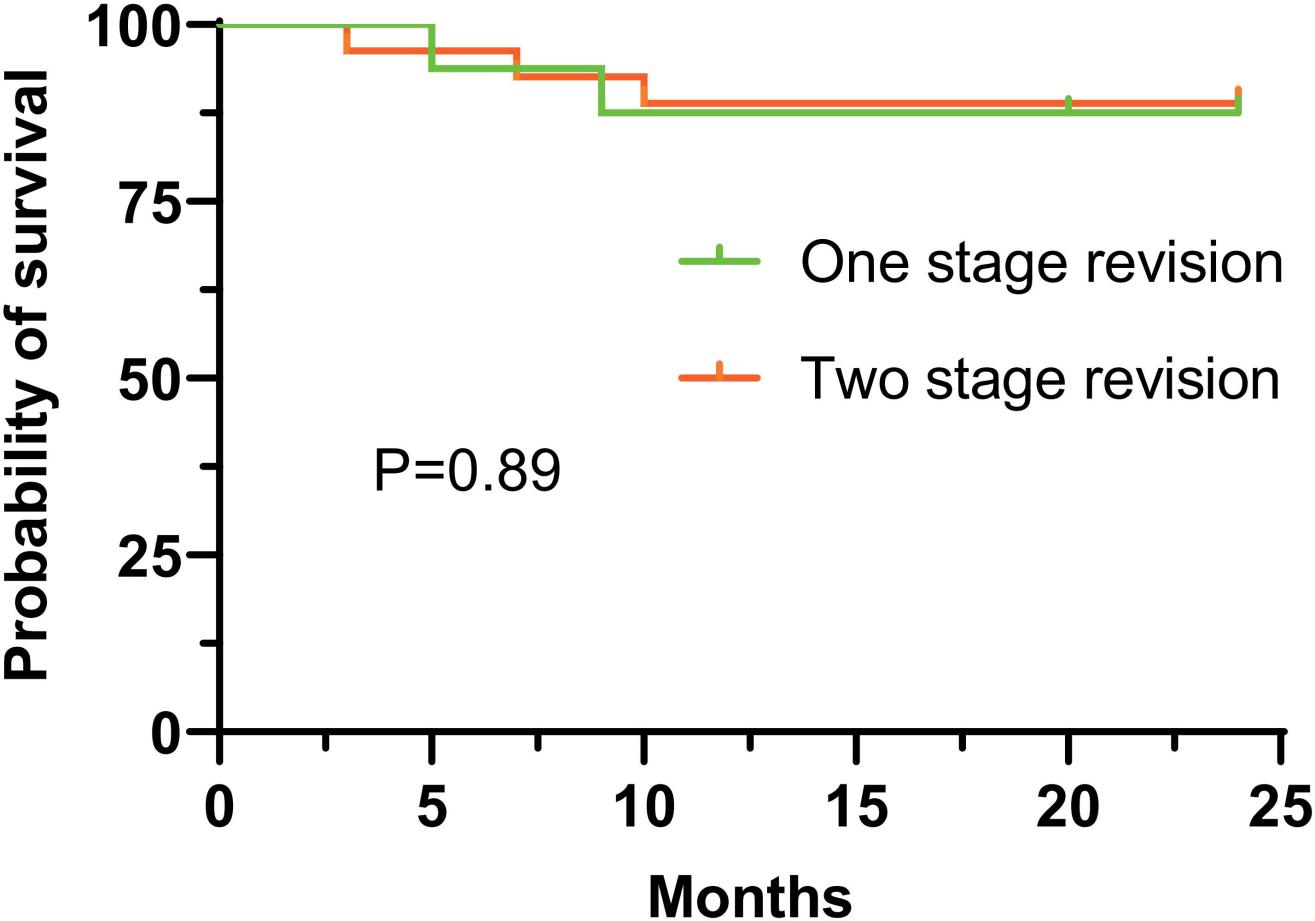
2-year survival rates after one- vs two-stage revision for chronic PJI.

All patients were followed up postoperatively, with a mean follow-up duration of 29.5 ± 4.5 months. Regarding the primary outcome, 2 cases of infection recurrence were observed in the one-stage revision group, resulting in an infection control rate of 87.5% (14/16). In the two-stage revision group, 3 cases of infection recurrence occurred, yielding an infection control rate of 88.9% (24/27). Comparison of infection control rates between the two groups showed no statistically significant difference (P = 0.89) (**Figure 1**).

### Case examples

**Figure 2:**
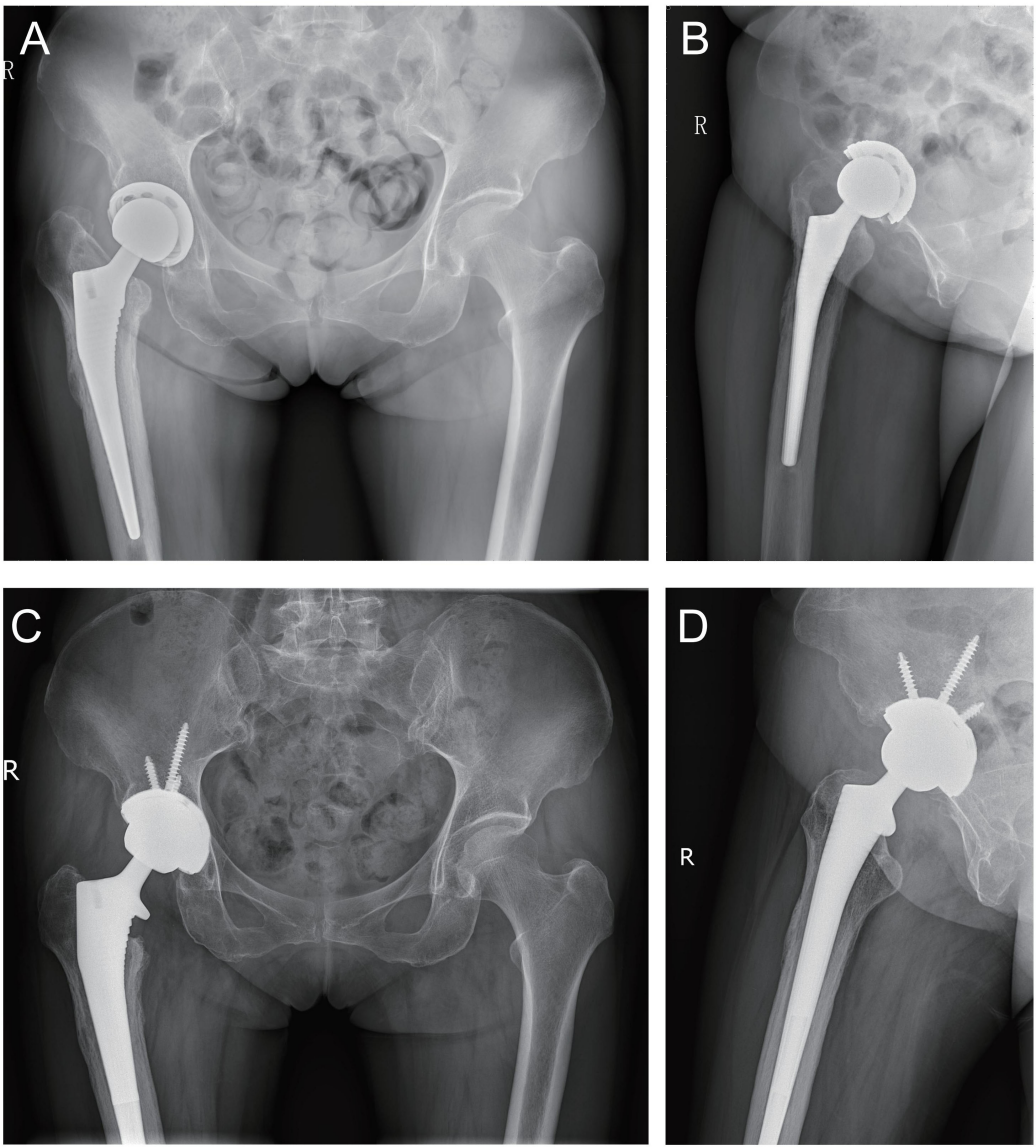
**(A, B)** represent the anteroposterior pelvic and lateral hip radiographs before revision surgery; **(C, D)** represent the anteroposterior pelvic and lateral hip radiographs 2 years after one-stage revision surgery.

**Figure 3:**
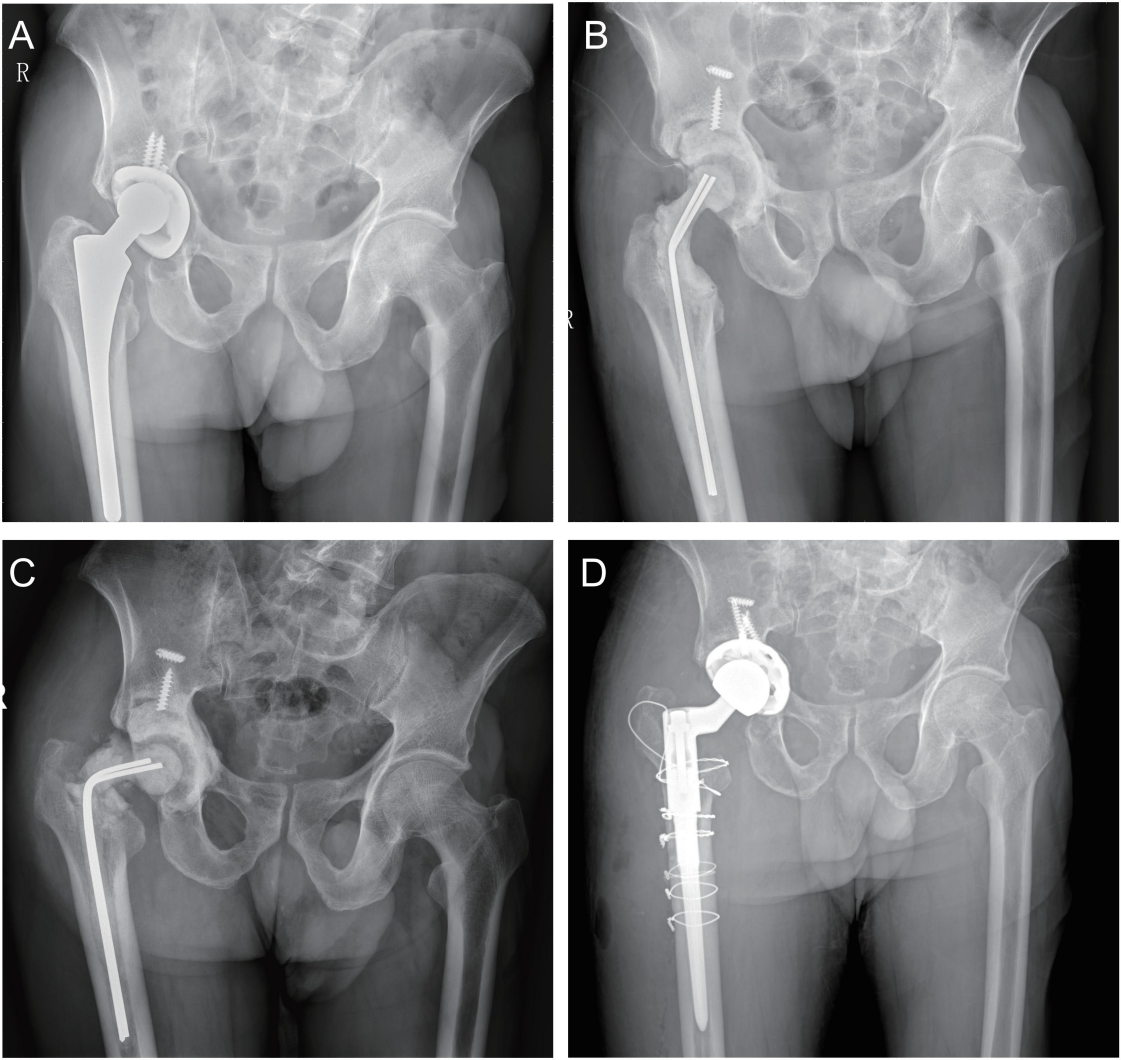
**(A)** postoperative anteroposterior pelvic radiograph; **(B)** anteroposterior pelvic radiograph 3 days after cement spacer implantation; **(C)** anteroposterior pelvic radiograph showing spacer fracture; **(D)** anteroposterior pelvic radiograph 2 years after two-stage revision.

**One Stage Revision:** A 62-year-old female experienced persistent pain and limited function 5 months after primary total hip arthroplasty. Her ESR was 65 mm/h and CRP was 59 mg/L. Preoperative cultures showed *Staphylococcus aureus* infection without sinus tract formation. She underwent one-stage revision surgery with complete debridement and simultaneous prosthetic reconstruction. No infection recurrence was observed during follow-up, inflammatory markers returned to normal, and hip function improved significantly (**Figure 2**). Two Stage Revision: A 68-year-old male developed joint pain 3 years after primary total hip arthroplasty. His ESR was 78 mm/h, CRP was 34 mg/L, and joint aspirate culture showed methicillin-resistant *Staphylococcus aureus* (MRSA). He underwent a two-stage revision. In the first stage, the prosthesis was removed, debridement was performed, and an antibiotic-loaded bone cement spacer was placed. Eighty-three days later, the spacer fractured, and a second hip revision surgery was performed. By the final follow-up, infection was controlled, and hip function had improved significantly compared to preoperative levels (**Figure 3**).

## Discussion

This study conducted a retrospective cohort analysis comparing the short-term outcomes of one-stage and two-stage revision surgeries for chronic PJI. The results indicated that, with preoperative identification of the pathogen and strict patient selection criteria, one-stage revision surgery achieved an infection control rate comparable to that of two-stage revision surgery at 2 years postoperatively, while also demonstrating certain advantages in joint function recovery. These findings suggest that one-stage revision surgery is a feasible and effective option for patients with PJI when the pathogen is clearly identified, aligning with recent trends in reevaluating the effectiveness of one-stage revision.

Two-stage revision has long been considered the “gold standard” for treating chronic PJI, with infection control rates typically ranging from 85% to 100% (Kini et al., 2016; Lazic et al., 2021; Kildow et al., 2022). However, recent studies have reported that one-stage revision can achieve similar infection control outcomes (van den Kieboom et al., 2021; Qin et al., 2024). In this study, the infection control rate for the one-stage revision group was 87.5%, and for the two-stage revision group, it was 88.9%. The difference between the two groups was not statistically significant, which is consistent with previous reports, further supporting the reasonable role of one-stage revision in the treatment of chronic PJI.

Regarding joint function recovery, this study found that the one-stage revision group showed a significantly greater improvement in joint scores at 24 months postoperatively compared to the two-stage revision group. Previous studies have suggested that in two-stage revision, the use of a bone cement spacer during the interval between surgeries may limit joint mobility, cause muscle atrophy, and lead to pain, all of which could negatively affect final functional recovery (Limtong et al., 2021; Naoum et al., 2024). In contrast, one-stage revision, through a single surgical procedure, allows for earlier weight-bearing and functional rehabilitation (Ghosh et al., 2025). The results of this study are consistent with these findings, suggesting that one-stage revision may have a potential advantage in improving functional outcomes for patients.

In terms of complications, this study found no significant difference in the incidence of complications within 90 days postoperatively between the two groups. The major complications in the one-stage revision group were wound healing issues and hematoma, while the two-stage revision group had complications including spacer fracture and superficial wound infection. Previous literature has reported that, due to the longer treatment duration and multiple surgeries, two-stage revision may carry an increased risk of perioperative complications (Sun et al., 2025; Thomas et al., 2025). Although no significant differences were observed in this study, the occurrence of spacer-related complications in the two-stage revision group suggests that this strategy still poses unique risks during the functional limitation phase, requiring enhanced monitoring and management in clinical practice.

The selection of the surgical strategy primarily depended on whether definitive microbiological evidence and antimicrobial susceptibility results were obtained preoperatively, which was the key criterion differentiating the two groups. Regarding temporal trends, one-stage revision accounted for approximately 20-30% of cases from 2018 to 2020, increasing to 45-50% from 2021 to 2023. This shift partly reflected increased confidence of the senior surgeon in one-stage revision, as early cases demonstrated better infection control outcomes during follow-up. This study has several limitations. As a single-center, retrospective study with a small sample size, the non-randomized design introduces the risk of selection bias and residual confounding. Although strict inclusion criteria, baseline matching, and standardization by a single surgeon were implemented, unmeasured confounders may still exist. The surgical strategy was based on preoperative microbiological evidence rather than random allocation. Therefore, the findings should be interpreted with caution, and multicenter, prospective randomized trials are needed for validation.

## Conclusion

The results of this study indicate that for patients with chronic PJI and clearly identified pathogens, one-stage revision surgery demonstrated similar infection control rates and safety outcomes compared to two-stage revision during short-term follow-up, with an additional advantage in postoperative joint function recovery. This treatment strategy may effectively reduce the need for a second surgery, shorten the overall treatment period, and thus alleviate the patient’s pain and economic burden. Therefore, this study suggests that one-stage revision may be a viable alternative for carefully selected patients with identified pathogens. Future studies should further validate the long-term efficacy and applicability of this treatment approach.

## Data Availability

The datasets presented in this article are not readily available because no data. Requests to access the datasets should be directed to qijinwang1940@163.com.
